# Vascular biomarkers and ApoE4 expression in mild cognitive impairment and Alzheimer's disease

**DOI:** 10.3934/Neuroscience.2018.2.148

**Published:** 2018-06-16

**Authors:** Diana C. Oviedo, Hector Lezcano, Ambar R. Perez, Alcibiades E. Villarreal, Maria B. Carreira, Baltasar Isaza, Lavinia Wesley, Shantal A. Grajales, Sara Fernandez, Ana Frank, Gabrielle B. Britton

**Affiliations:** 1 Universidad Católica Santa María La Antigua (USMA), Panamá; 2 Facultad de Medicina, Universidad de Panamá, Panamá; 3 Centro de Neurociencias y Unidad de Investigación Clínica, Instituto de Investigaciones Científicas y Servicios de Alta Tecnología (INDICASAT AIP), Panamá; 4 Servicio de Radiología, Complejo Hospitalario Arnulfo Arias Madrid, Caja del Seguro Social, Panamá; 5 Departmento de Psicología Básica II (Procesos Cognitivos), Facultad de Psicología, Universidad Complutense de Madrid, Madrid, España; 6 Servicio de Neurología, Hospital Universitario La Paz, Madrid, España

**Keywords:** aging, cognition, atherosclerosis, intima-media thickness, stenosis, Latin America, Panama

## Abstract

Vascular pathology and genetic markers such as apolipoprotein E allele ε4 (ApoE ε4) are risk factors for the progression from mild cognitive impairment (MCI) to Alzheimer's disease (AD). In Panama, a high prevalence of vascular risk factors and an increase in the aging population, generate the need to investigate biomarkers using specific, sensitive, non-invasive and cost-efficient methods that could be used in primary care. The main objective of this study was to explore the association between vascular biomarkers such as intima-media thickness (IMT) and stenosis, ApoΕ ε4 and cognitive function in a sample of older adults, including healthy controls (*n* = 41), MCI (*n* = 33), and AD (*n* = 12). A descriptive and cross-sectional study was conducted. Participants were part of the Panama Aging Research Initiative (PARI), the first prospective study in aging in Panama. Assessments included a neuropsychological battery, ApoΕ ε4 genotyping and a Doppler ultrasound of the left carotid artery to examine the presence of vascular risk factors. Neuropsychological tests were combined to form six cognitive domains: Global cognition, language, visuospatial abilities, learning and memory, attention and executive functions. Multivariable analyses (using age, education, and ApoE ε4 expression as covariates) were conducted. Participants with increased IMT showed poorer performance in memory and those with carotid stenosis showed poorer performance in language, visuospatial abilities and attention, independent of age, education or ApoΕ ε4 expression. The results support the use of vascular markers in cognitive assessments of aged individuals.

## 1. Introduction

Multiple reports indicate that individuals over 60 years old are the fastest growing group on earth [1]. A reduced mortality rate and the advances in medicine in the last decades have resulted in an increase in life expectancy and in the elderly population [2]. As people age they are more likely to develop chronic diseases such as mild cognitive impairment (MCI) and Alzheimer's disease (AD) [3]. The Latin American and Caribbean (LAC) region is experiencing one of the fastest aging rates [4], and as a result the prevalence of dementia and MCI has increased causing economic, social and public health burdens. Therefore, one of the main objectives in AD research is to identify and study risk factors that could contribute to the discovery of specific, sensitive, non-invasive and cost-efficient methods that could be used in primary care for early detection of AD.

Numerous studies have shown that vascular pathologies such as cardiovascular disease are risk factors for AD and MCI [5],[6]. Carotid atherosclerosis is a chronic inflammatory disorder characterized by the accumulation of plaques in the walls of large and medium arteries [7],[8]. Atherosclerosis is a risk factor for cerebrovascular diseases such as stroke, silent brain micro infarcts and brain hemorrhages, causing white matter lesions, neural dysfunction and cognitive impairment [9],[10].

During the process of aging, arteries undergo changes such as thickening of the intima-media and changes in the size and thickness of veins and arteries. The intima refers to the internal portion of the artery formed by an endothelium. The media or middle tunic is the middle layer of the artery. The distance between the intima and the media is known as the intima-media thickness (IMT) [11]. Several authors have reported associations between vascular risk factors such as IMT and stenosis and deficits in cognitive functions, although results have been inconsistent. An increased IMT can lead to a poor performance in memory and other cognitive functions such as language, attention, executive functions and psychomotor abilities [12]–[14]. However, other studies have not found such associations [15],[16]. Therefore, the evaluation of these vascular markers can be a crucial step in identifying elderly individuals at risk of developing cognitive impairment.

Among genetic risk factors, apolipoprotein E ε4 (ApoE ε4) has been shown to be the strongest risk factor for AD [17],[18]. ApoE ε4 expression increases the risk of developing dementia three to ten times [19]. ApoE ε4 and increased IMT have also been associated with cardiovascular disease [20]. Studies have shown that individuals with at least one copy of ApoΕ ε4 have a higher prevalence of cortical microinfarcts, atherosclerotic pathology, hemorrhages, thrombosis, cerebral amyloid angiopathy, cerebrovascular ischemia, pulsatility, hypertension, diabetes, among others [21]. In addition, ApoΕ ε4 is associated with an altered mechanism of cerebral circulation in older adults [22]. Evidence has stated that ApoE ε4 and vascular risk factors combined aggravate cognitive impairment [21] and their assessment can help in the understanding of the progression of MCI to AD [12].

To date, there are numerous studies focusing on the risk factors associated with cerebrovascular health and cognition in the elderly population, nevertheless most of this research has been carried out in developed countries. In the LAC region, the prevalence of vascular chronic diseases is increasing [23],[24]. In Panamá, prevalence studies have shown that cardiovascular diseases are the leading cause of death [25]. To our knowledge, there are no studies that focus on the relationship between vascular pathologies, ApoE ε4 and cognitive impairment in LAC countries. In the present study, we examined the association between carotid IMT and stenosis, ApoE ε4 and cognitive function in a sample of elderly adults in Panama. Based on evidence from previous studies, we expected that vascular risk factors and ApoE ε4 would influence performance in specific cognitive domains.

## 2. Methods

### 2.1. Participants

Data were analyzed from 86 participants of the Panama Aging Research Initiative (PARI) cohort [26],[27]. Volunteers were recruited from the outpatient geriatric services of the Social Security (CSS), the largest public hospital located in Panama City. Inclusion criteria encompassed being 65 years or older, having received the baseline cognitive assessment, willingness to participate in the follow-up visit and having signed the informed consent. Exclusion criteria consisted of any medical condition that interfered with the person's ability to attend the evaluation, illiteracy and participation in an ongoing clinical study at the time of enrollment. The protocol was approved by the Bioethics Committee of the CSS. Participants who were eligible for the study were explained the purpose of the study, the procedure, what was expected from them and then signed informed consent forms. Confidentiality was not breached in accordance with the principles of the Declaration of Helsinki (1964).

Participants underwent a standardized assessment protocol that included an interview to obtain information on sociodemographic characteristics, medical history, functional status and risk factors. A subsample of individuals (*n* = 70) underwent Doppler sonography to estimate the presence of vascular risk factors. A non-fasting blood sample was obtained to genotype for ApoE ε4. Interviews and evaluations were conducted in Spanish and reviewed by physicians, medical students and graduate students. Clinical data, medical records and imaging were examined by experienced clinicians. Approximately 17 months (*M* = 16.8 months, *SD* = 3.4) after baseline assessments participants underwent a follow-up interview, cognitive testing and assessment of functional status, subjective health status and presence of depressive symptoms. Interviews and neuropsychological evaluations were completed in a single visit (1.5–2 hours) and were conducted in Spanish by students and neuropsychologists.

### 2.2. Variables and measurements

#### 2.2.1. Clinical and neuropsychological assessment

The neuropsychological test battery included measures of six cognitive domains: 1) global cognition (Mini-Mental State Examination, MMSE) [28]; 2) attention (Digit Span forward [29] and Trail Making Test part A [30]); 3) executive function (Trail Making Test part B [30] and Digit Span Backward [29]); 4) memory (10 word free recall immediate and delayed list [31]); 5) language (Boston Naming [32] and Semantic Verbal Fluency [33]); and 6) visuospatial abilities (Clock Drawing copy version [34] and Poppelreuter Test [35]). Basic and instrumental activities of daily living were assessed with the Lawton and Brody Instrumental Activities of Daily Living Scale [36] and Functional Activities Questionnaire (FAQ) [37]. Depression was assessed with the Spanish version of the 30-item Geriatric Depression Scale (GDS-30) [38], and health subjective status was evaluated using the European Quality of Life EuroQol Health Questionnaire (EGQ-5D-3L) [39]. Stages of cognitive function were rated according to the Global Deterioration Scale (GDetS) [40]. All information was reviewed by a consensus committee who diagnosed participants with AD, MCI or no cognitive impairment (normal controls; NC). Participants were included as controls if they performed within normal limits in the neuropsychological assessment and scored ≤ 10 in the GDS-30 (below the threshold for symptoms of depression). MCI diagnosis was based on core clinical criteria [41] and required deficits in at least one cognitive domain, independence in activities of daily living and a rating of GDetS ≤ 3. Diagnosis of AD was based on NINCDS-ADRDA [42] criteria and required evidence of impairments in memory and at least one other cognitive domain, impairments in everyday social and/or work-related activities, and a GDetS score of four or higher (range 1–7).

#### 2.2.2. ApoE ε4 genotyping

For ApoE genotyping, DNA samples were obtained from whole blood leukocytes (EDTA plasma collection tubes) using QIAmp DNA Mini Kit (Qiagen) according to manufacturer recommendations. ApoE genotyping was conducted according to standardized PCR procedures [43].

#### 2.2.3. Ultrasound assessment of carotid IMT and stenosis

High resolution B-mode ultrasonography (LOGIQ e GE Medical Systems, China) with 7.5 MHz high frequency linear transducer was used to measure the volume and speed of blood flow and IMT and stenosis in the left carotid artery. The exam was conducted while the participant was lying in a supine position with the head slightly rotated to 45^°^ from the examiner, first from a cross-sectional view, starting from the base of the neck up to the bifurcation in the internal carotid and external carotid arteries. IMT was measured at the level of the distal portion of the left common carotid artery and was defined as the distance (in millimeters) between the leading edges of the lumen-intima and media-adventitia interfaces of the arterial wall. A cut-off value of 0.9 mm was considered abnormal thickening [44]. In addition, blood flow velocities and the presence of atheromatous plaques were evaluated. Carotid artery stenosis was determined using values of the peak systolic velocity (PSV) as follows: (1) normal when PSV < 125 cm/s without visible plaque or intimal thickening; (2) < 50% stenosis when PSV < 125 cm/s and visible plaque or intimal thickening; (3) 50–69% stenosis when PSV 125–230 cm/s and visible plaque; (4) ≥ 70% stenosis to near occlusion when PSV > 230 cm/s and visible plaque and lumen narrowing are seen; (5) near occlusion when there was a markedly narrowed lumen; and (6) total occlusion when there was no detectable lumen [45]. Values were then dichotomized into absence (normal) or presence of stenosis (all other values).

### 2.3. Statistical analysis

Analyses were performed using SPSS 21.0 (Armonk, NY: IBM Corp). First, demographic and clinical characteristics were examined using descriptive statistics. Univariable one-way analysis of variance (ANOVA) and chi square (*Χ^2^*) tests were applied to continuous and categorical variables, respectively, and post hoc comparisons were conducted with Bonferroni tests. Neuropsychological test scores were converted to *z* scores, then summed and averaged to calculate an average *z* score in six cognitive domains: Global cognition, language, visuospatial, memory, executive function and attention. Cognitive performance between groups was compared using analysis of covariance (ANCOVA) and using age, education and ApoE ε4 expression as covariates. Values of *p* < 0.05 were considered statistically significant.

Separate multivariable analyses of covariance (MANCOVA) were conducted in order to establish the influence of vascular risk factors and ApoE ε4 on cognitive performance across diagnostic groups. Covariates included age, education and ApoE ε4 expression.

## 3. Results

### 3.1. Sample characteristics

Diagnostic groups did not differ in sex, education, depressive symptoms, subjective health status, stenosis or ApoE ε4 expression (Table 1). The percentage of the AD group with IMT ≥ 0.9 mm was greater than the control and MCI groups, and the MCI group also differed from controls. As expected, groups differed in performance across all cognitive domains, independent of age, education and ApoE ε4 expression, although MANCOVA revealed greater deficits in global cognition in the AD group in the presence of ApoE ε4.

### 3.2. Association between IMT, stenosis, ApoE ε4, and cognitive performance

Tables 2 and 3 summarize the results of 2 × 3 MANCOVAs combining diagnostic groups and vascular markers. There was no significant interaction between diagnostic groups and IMT (Table 2) or stenosis (Table 3). Therefore, IMT groups were examined independent of diagnosis. This analysis showed that IMT values equal to or greater than 0.9 mm were associated with a lower performance in the learning and memory domain [*F*(1,65) = 9.03, *p* = 0.004] independent of age, education and ApoE ε4 expression (Table 4). Also, there was a tendency for poorer performance in the language and visuospatial domains (*p*s < 0.07). When participants were divided into those with and without stenosis (Table 5), stenosis was significantly associated with language [*F*(1,65) = 12.81, *p* = 0.001], visuospatial [*F*(1,65) = 7.72, *p*= 0.007], memory [*F*(1,65) = 11.24, *p* = 0.001], and attention [*F*(1,65) = 5.08, *p* = 0.028] deficits.

**Table 1.** Demographic characteristics

		Normal control (*n* = 41)	MCI (*n* = 33)	AD (*n* = 12)	*Test statistic*	*p*
Years of Study		10.7 (4.9)	9.5 (4.0)	7.9 (4.4)	*F*(2,83) = 2.0	0.141
Age		76.6 (5.6)	79.2 (7.8)	82.4 (7.9)^a^	*F*(2,83) = 3.0	0.030
% female sex		31 (75.6%)	21 (63.6%)	10 (83.3%)	χ^2^ (2) = 2.2	0.336
BMI		26.3 (4.9)	24.1 (4.4)	23.3 (6.1)	*F*(2,83) = 2.6	0.080
EQ-5D-3L		76.6 (18.6)	70.9 (22.7)	80.5 (17.1)	*F*(2,82) = 1.2	0.303
FAQ		1.2 (2.7)^b^	2.1 (2.7)	18.3 (6.6)^a^	*F*(2,83) = 12.4	0.000
Functionality Index		0.9 (0.2)^b^	0.9 (0.1)	0.4 (0.2)^a^	*F*(2,83) = 78.7	0.000
GDetS		1.5 (0.6)^b^	2.5 (0.5)^a^	4.9 (0.9)^a^	*F*(2,83) = 9.5	0.000
GDS-30		5.6 (4.9)	7.5 (5.2)	9.3 (5.9)	*F*(2,83) = 2.7	0.071
% IMT ≥ 0.9 mm		11/32 (34.4%)	15/27 (55.6%)	9/11 (81.8%)	χ (2) = 7.9	0.019
% Stenosis		8/32 (25.0%)	11/27 (40.7%)	7/11 (63.6%)	χ (2) = 5.5	0.065
ApoE ε4		9/40 (22.5%)	11/32 (34.4%)	6/12 (50.0%)	χ (2) = 3.6	0.170
Global Cognition		0.5 (0.4)	0.2 (0.5)	−1.2 (1.3)^ab^	*F*(2,77) = 25.7	0.000
Language		0.4 (0.5)^b^	0.04 (0.7)	−1.0 (0.8)^ab^	*F*(2,77) = 18.4	0.000
Visuospatial		0.4 (0.3)	0.1 (0.5)	−1.1 (1.4)^ab^	*F*(2,77) = 18.5	0.000
Memory		0.6 (0.6)^b^	−0.3 (0.5)	−0.9 (0.7)^ab^	*F*(2,77) = 32.0	0.000
Attention		0.3 (0.5)^b^	−0.07 (0.6)	−0.7 (0.8)^ab^	*F*(2,77) = 8.0	0.001
Executive Function		0.4 (0.6)^b^	−0.2 (0.7)	−0.4 (0.4)^a^	*F*(2,77) = 7.5	0.001

Functionality Index: Number of activities on which the participant was independent divided by the total number of activities assessed; ApoE ε4: % ApoE with at least one copy of ε4 allele. Control, MCI and AD groups were compared using ANOVA for continuous variables and Pearson chi-square for categorical variables. ANOVA post hoc comparisons were conducted with Bonferroni tests. *p* < 0.05 was considered statistically significant. ^a^Statistically different from control group. ^b^Statistically different from MCI group. This table also describes the ANCOVA comparing z-scores for each cognitive domain between control, MCI and AD groups, controlling for age, education and ApoE4. The comparison was considered significant when *p* < 0.05.

**Table 2.** Association between IMT and diagnostic groups for each cognitive domain

Cognitive Domains	NC (*n* = 32)		MCI (*n* = 27)		AD (*n* = 11)		*F*(2,61)	*p*	η_p_^2^
	< 0.9 IMT	≥ 0.9 IMT	< 0.9 IMT	≥ 0.9 IMT	< 0.9 IMT	≥0.9 IMT			
Global Cognition	0.6 (0.2)	0.4 (0.4)	0.1 (0.4)	0.2 (0.6)	−1.1 (2.7)	−1.4 (1.2)	0.5	0.594	0.02
Language	0.5 (0.4)	0.5 (0.7)	0.1 (0.8)	−0.1 (0.6)	−0.6 (0.5)	−1.2 (0.8)	0.2	0.794	0.01
Visuospatial	0.4 (0.2)	0.4 (0.3)	0.2 (0.5)	0.04 (0.6)	−0.3 (1.2)	−1.5 (1.4)	1.9	0.148	0.06
Memory	0.9 (0.5)	0.2 (0.5)	−0.1 (0.6)	−0.3 (0.6)	−0.9 (0.1)	−0.9 (0.8)	1.3	0.273	0.04
Attention	0.4 (0.5)	0.3 (0.6)	−0.1 (0.5)	−0.2 (0.6)	−0.9 (0.2)	0.9 (0.8)	0.2	0.822	0.01
Executive Function	−0.4 (0.5)	−0.5 (0.6)	−0.5 (0.6)	−0.1 (0.6)	−0.8 (0.6)	−0.4 (0.3)	0.4	0.692	0.01

This table summarizes the average z scores for each cognitive domain. Statistics describe the MANCOVA comparing group IMT < 0.9 and IMT ≥ 0.9 within each diagnostic group for each cognitive domain, controlling for age, education and ApoE4. MANCOVA post hoc comparisons were conducted with Bonferroni tests. *p* < 0.05 was considered statistically significant.

**Table 3.** Association between stenosis and diagnostic groups for each cognitive domain

Cognitive Domains	NC (*n* = 32)		MCI (*n* = 27)		AD (*n* = 11)		*F*(2,61)	*p*	η_p_^2^
	No stenosis	Stenosis	No stenosis	Stenosis	No stenosis	Stenosis			
Global cognition	0.6 (0.3)	0.4 (0.4)	0.1 (0.5)	0.2 (0.5)	−1.4 (1.6)	−1.3 (1.3)	0.3	0.715	0.01
Language	0.6 (0.4)	0.4 (0.8)	0.1 (0.6)	−0.2 (0.7)	−0.9 (0.6)	−1.1 (0.9)	0.2	0.829	0.01
Visuospatial	0.4 (0.2)	0.4 (0.3)	0.2 (0.5)	0.02 (0.6)	−0.7 (0.9)	−1.6 (1.6)	2.5	0.094	0.08
Memory	0.8 (0.5)	0.2 (0.6)	−0.1 (0.6)	−0.5 (0.5)	−0.9 (0.1)	−0.9 (0.9)	0.9	0.400	0.03
Attention	0.3 (0.5)	0.5 (0.6)	−0.01 (0.6)	−0.4 (0.5)	−0.8 (0.1)	−0.9 (0.9)	1.0	0.371	0.03
Executive Function	0.4 (0.6)	0.6 (0.4)	0.3 (0.7)	−0.3 (0.6)	−0.6 (0.4)	−0.4 (0.3)	0.1	0.932	0.00

This table summarizes the average z scores for each cognitive domain. Statistics describe the MANCOVA comparing the group with no stenosis and with stenosis within each diagnostic group for each cognitive domain, controlling for age, education and ApoE4. MANCOVA post hoc comparisons were conducted with Bonferroni tests. *p* < 0.05 was considered statistically significant.

**Table 4.** Association between IMT and cognitive domains

Cognitive Domains	< 0.9 IMT (*n* = 35)	≥ 0.9 IMT (*n* = 35)	*F*(1,65)	*p*	η_p_^2^
Global Cognition	0.3 (0.7)	−0.2(1.0)	1.7	0.199	0.03
Language	0.3(0.6)	−0.2 (0.9)	3.5	0.065	0.05
Visuospatial	0.3 (0.4)	−0.2 (1.1)	4.0	0.051	0.06
Memory	0.5 (0.8)	−0.3 (0.7)	9.0	0.004	0.12
Attention	0.2 (0.6)	−0.2 (0.8)	2.7	0.107	0.04
Executive Function	0.02 (0.7)	0.0 (0.6)	0.8	0.367	0.01

This table summarizes the average z scores for each cognitive domain. Statistics describe the ANCOVA comparing group IMT < 0.9 and ≥ 0.9 for each cognitive domain, controlling for age, education and ApoE4. ANCOVA post hoc comparisons were conducted with Bonferroni tests. *p* < 0.05 was considered statistically significant.

**Table 5.** Association between stenosis and cognitive domains

Cognitive Domains	No stenosis (*n* = 44)	Stenosis (*n* = 26)	*F*(1,65)	*p*	η_p_^2^
Global Cognition	0.2 (0.8)	−0.1 (1.0)	2.2	0.148	0.03
Language	0.3 (0.7)	−0.3 (0.9)	12.8	0.001	0.17
Visuospatial	0.2 (0.5)	−0.3 (1.2)	7.7	0.007	0.11
Memory	0.3 (0.8)	−0.4 (0.7)	11.2	0.001	0.15
Attention	0.1 (0.6)	−0.3 (0.8)	5.1	0.028	0.07
Executive Function	0.03 (0.7)	−0.03 (0.6)	0.0	0.987	0.00

This table summarizes the average z scores for each cognitive domain. Statistics describe the ANCOVA comparing no stenosis versus stenosis groups for each cognitive domain, controlling for age, education and ApoE4. ANCOVA post hoc comparisons were conducted with Bonferroni tests. *p* < 0.05 was considered statistically significant.

## 4. Discussion

The main objective of this study was to explore the impact of vascular biomarkers such as IMT and stenosis on cognitive function in aged adults diagnosed with MCI or AD. Initially participants were assessed with a cognitive test battery, and as expected, groups performed differently across cognitive domains. The tests used in this study are common in AD research and diagnosis and have yielded similar results [46],[47]. In addition, the combination of tests to form composite scores has generated comparable results in several studies [48],[49] where the most frequently studied domains were attention, executive functions, global cognition, processing speed, episodic memory, verbal abilities and visuospatial abilities [50]. Our findings confirmed that participants with AD with at least one copy of ApoE ε4 had a significantly lower performance in global cognition [26].

Vascular markers were examined to identify their association with cognitive function. The results showed that there was no significant effect of IMT and stenosis when they are examined together with group diagnosis. However, when diagnosis was not considered, having an IMT ≥ 0.9 mm was associated with worse performance in memory; likewise the presence of carotid stenosis was related to worse performance in language, visuospatial abilities, memory and attention. These results were independent of age, education or ApoE ε4 expression.

Several studies have found a positive relationship between IMT and memory deficits [51]. Longitudinal studies that included older adults without a diagnosis of vascular pathologies or dementia, found that the higher the IMT, the lower the performance on memory tasks [51],[52]. Memory alteration can be a preclinical manifestation of dementia, so the association between the vascular marker and the memory deficit can play a decisive role in establishing which subjects have a higher risk of progressing towards a more pronounced stage of cognitive deterioration. Consistent with our findings, others have found that vascular alterations were associated with lower cognitive performance in memory, attention, processing speed and executive function [53],[54]. In contrast, other studies found no relationship between IMT and the memory domain, although greater IMT values were associated with deficits in executive functions and global cognition [48]. Likewise, as we observed, cognitive performance in multiple cognitive functions has been shown to be lower in subjects with stenosis [55],[56]. In studies that compared cognitive performance of subjects with and without stenosis, subjects with stenosis had a worse performance in attention, psychomotor speed, memory, motor skills [57], visuospatial abilities and language [58],[59].

There are different mechanisms that could explain the association between vascular alterations and cognitive impairment. First, changes in the arteries such as such as luminal narrowing or IMT thickening can reduce blood circulation and interrupt the flow of nutrients to the brain. As a result, different cognitive domains may be affected [5],[60]. Also, it has been reported that an increase in IMT is a consequence of other vascular pathologies such as hypertension and atherosclerosis that may be related to brain changes, such as atrophy and white matter lesions, which also alter cognition [15],[61]. Specifically, atrophy of temporal lobe structures is associated with difficulties learning and recovering information [62],[63].

This study had several limitations, one of which was the small number of participants who were diagnosed with AD. A greater sample size would clarify further potential interactions among the variables examined. Also, the study was cross-sectional so we cannot draw causal inference about the variables measured and cognitive function. Another potential limitation involves the accuracy of AD and MCI diagnosis. Evidence has shown that the clinical diagnosis of AD has an accuracy of 70–90% [64] and the diagnosis of MCI is even more complex due do its mixed etiology that can be influenced by multiple factors. As such, our results should be interpreted with these limitations in mind. Each of these limitations is being addressed in ongoing studies. Study strengths include providing the first report of cognitive impairment associated with vascular markers and ApoΕ4 in the LAC region. The association between these measures reveals the possibility of incorporating markers (based on their association with neuropsychological tests) at the level of primary care in order to have additional information that could help establish the risk factors for cognitive impairment. Currently, no biological marker is used to detect individuals at risk of cognitive impairment in local public health facilities.

## 5. Conclusion

In Panama, research and policies focused on the health of older adults continue to be scarce. One of the main problems is that research on aging and associated conditions is insufficient making it difficult to develop biomarkers for diseases associated with cognitive impairment. On the other hand, there is a lack of adequate, consistent and timely diagnoses, especially in primary care. Thus our study contributes to the understanding of risk factors among Hispanics both within and outside LAC. Our results indicate that including vascular markers in the assessment of older adults can provide a non-invasive tool that can facilitate early diagnosis of age-related impairments. These results support the notion that regardless of diagnosis, vascular pathologies are associated with worse performance in specific domains, which could serve to guide assessments in primary care.
